# Radiotherapy for glioma in the AI era: current applications and future prospects

**DOI:** 10.3389/fonc.2025.1673752

**Published:** 2025-09-11

**Authors:** Xin Wang, Zhaoyang Qi, Qin Zeng, Dongling Gu, Tianliang Li

**Affiliations:** ^1^ Department of Pediatric Surgery, Zigong First People’s Hospital, Zigong, China; ^2^ Zigong First People’s Hospital, Zigong, China; ^3^ STU-CUHK Joint Shantou International Eye Center, Shantou, China

**Keywords:** artificial intelligence, glioma, radiotherapy, prospects, clinical practice

## Abstract

Gliomas are primary central nervous system tumors characterized by a high recurrence rate and poor prognosis, especially in high-grade forms such as glioblastoma (GBM). Radiotherapy remains a cornerstone in glioma management, particularly following surgical resection. Recent advancements in technology—including intensity-modulated radiotherapy (IMRT), proton therapy, carbon-ion radiotherapy, intraoperative radiotherapy, and ultra-high dose rate FLASH radiotherapy—have improved treatment precision and tumor control. However, clinical challenges persist due to tumor heterogeneity, imaging limitations, and planning variability. In the era of artificial intelligence (AI), novel tools such as radiomics, deep learning, and predictive modeling are increasingly being integrated into glioma radiotherapy workflows. These AI-driven approaches have shown potential to enhance imaging interpretation, automate contouring, optimize treatment planning, and predict clinical outcomes. This review highlights the evolution of glioma radiotherapy, explores the emerging role of AI across various stages of radiotherapy, and discusses future directions for implementing personalized, adaptive, and data-driven strategies in clinical practice.

## 1 Introduction

Gliomas are solid tumors originating from glial cells of the central nervous system, with an annual incidence of approximately 5–8 per 100,000 individuals (1), and they represent the most frequently diagnosed intracranial neoplasms in pediatric populations (2). According to the fifth edition of the World Health Organization (WHO) classification of central nervous system tumors released in 2021, gliomas are categorized into grades I to IV (3). Grade IV glioblastoma (GBM) is the most aggressive form, and despite comprehensive multimodal therapies—including surgery, concurrent chemoradiotherapy, and adjuvant chemotherapy—the median overall survival remains less than two years, with a 5-year survival rate of approximately 10% (4). Radiotherapy is a critical component in the therapeutic management of glioma, particularly for controlling residual disease post-surgery (5, 6). Nonetheless, challenges such as imprecise target delineation and suboptimal dose distribution continue to hinder treatment outcomes. The rapid advancement of artificial intelligence (AI) has enabled its application across several stages of radiotherapy—including image processing, contour automation, treatment planning, and outcome prediction—creating new opportunities for enhancing precision and personalization (7, 8). This review provides an overview of current AI applications in glioma radiotherapy, examines key technical challenges, and outlines future prospects for clinical integration.

## 2 Role and limitations of radiotherapy in glioma treatment

**Figure 1:**
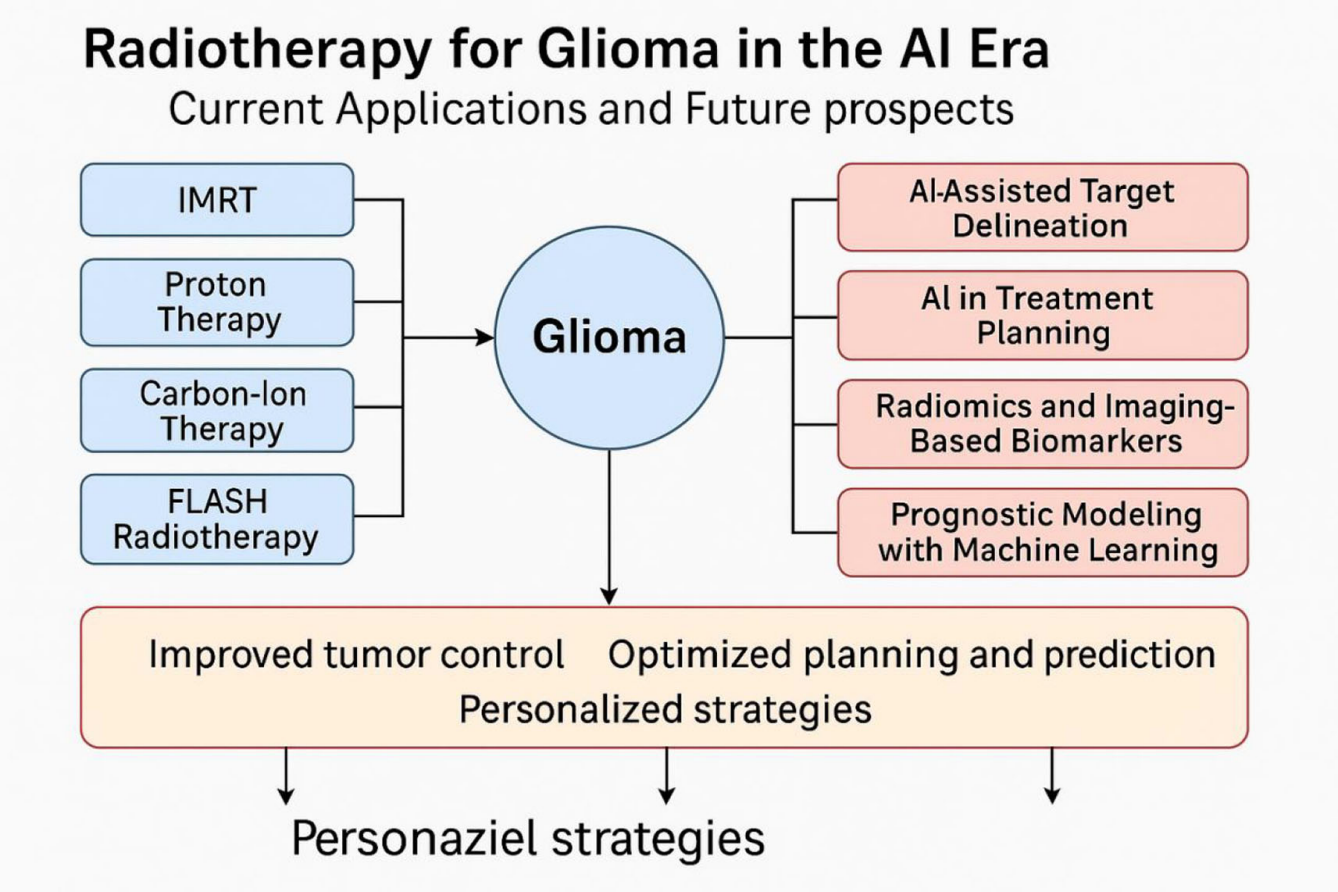
Overview of radiotherapy strategies and artificial intelligence applications in glioma treatment.

**Figure 1** illustrates the integration of conventional radiotherapy techniques with emerging AI applications in glioma management. The synergy between advanced physical delivery modalities (e.g., Intensity-Modulated Radiotherapy [IMRT], proton and carbon-ion therapy, and FLASH radiotherapy) and AI-driven technologies (e.g., auto-segmentation, treatment planning optimization, radiomics, and prognostic modeling) aims to enhance tumor control and enable truly personalized treatment strategies.

With the development of radiotherapy techniques, modalities such as IMRT, volumetric-modulated arc therapy (VMAT), proton therapy, carbon-ion therapy, and FLASH radiotherapy are increasingly being used in clinical practice (9–14). Enhancing local tumor control while minimizing damage to surrounding healthy tissue remains a central challenge in the radiotherapeutic management of gliomas.

Currently, the standard adjuvant radiotherapy protocol for high-grade gliomas (HGG) is the Stupp regimen (15, 16). This involves initiating fractionated radiotherapy approximately four weeks after surgery, delivering a total dose of 60 Gy in daily fractions of 1.8–2 Gy, concurrently with temozolomide (TMZ) chemotherapy at a dose of 75 mg/m². Approximately one month after completing concurrent chemoradiotherapy, patients proceed with six cycles of adjuvant TMZ chemotherapy.

For elderly patients or those with poor performance status, hypofractionated radiotherapy (e.g., 40 Gy in 15 fractions or 34 Gy in 10 fractions) can provide comparable efficacy to conventional fractionation.

Despite technological advances, therapeutic gains remain modest. Primary obstacles include: (1) intra- and inter-patient tumor heterogeneity, with diverse genetic drivers affecting radiosensitivity and recurrence patterns; (2) limitations in target delineation, as conventional MRI often fails to fully characterize infiltrative tumor margins, leading to under- or over-treatment; (3) static treatment planning, which cannot adapt to anatomical or pathological changes during therapy, such as edema or tumor shrinkage; and (4) variability in contouring and plan quality among clinicians and treatment centers.

These challenges collectively hinder the realization of precision radiotherapy and underscore the need for personalized and adaptive approaches.

### 2.1 AI in real-time adaptive planning

While radiotherapy has been a cornerstone in glioma treatment, traditional methods face significant limitations due to tumor heterogeneity, anatomical shifts, and edema changes during treatment (17). Real-time adaptive planning—the ability to dynamically adjust the treatment plan based on tumor changes during radiotherapy—has emerged as a promising avenue, and AI plays a critical role in facilitating this process (18).

AI can assist in real-time adaptive planning in several key ways:

Real-Time Imaging and Analysis: AI algorithms, particularly deep learning models, can process imaging data in real time, providing up-to-date tumor delineation and identifying changes in tumor volume or location (19). This ensures that the treatment plan is dynamically adjusted to account for tumor motion, edema, or anatomical shifts during radiotherapy sessions.

Treatment Adaptation: AI models can guide adjustments to the radiation dose distribution during radiotherapy, continuously optimizing the treatment plan based on updated tumor and organ-at-risk (OAR) positions (20). Reinforcement learning algorithms, for instance, can be used to learn from each radiotherapy session and make adjustments for future treatments.

Clinical Decision Support: Integrating AI with treatment delivery systems allows clinicians to receive real-time feedback on tumor changes (21). This feedback facilitates timely decisions on radiation dose adaptation, improving treatment precision and enhancing the effectiveness of radiotherapy, particularly for gliomas, where accurate tumor tracking and adaptability are crucial.

### 2.2 IMRT

IMRT has become a widely accepted standard for glioma treatment due to its ability to deliver highly conformal radiation doses to complex target volumes while sparing adjacent OARs such as the optic nerves, brainstem, and hippocampus. Through inverse planning algorithms and multileaf collimator (MLC) modulation, IMRT enhances dose conformity in irregularly shaped lesions typical of HGGs, especially those located near eloquent brain regions (22).

For WHO grade II high-risk low-grade glioma, Wang et al. (23) reported that both IMRT alone and in combination with TMZ significantly improved median progression-free survival (mPFS) and overall survival (mOS) compared with observation alone. Specifically, the mPFS was 59 months in the observation group, 82 months in the radiotherapy group, and not reached in the STUPP group; for OS, the median was 96 months in the observation group, while both RT and STUPP groups did not reach a median OS.

VMAT, a time-efficient evolution of IMRT, delivers intensity-modulated beams during continuous gantry rotation. By simultaneously varying gantry speed, dose rate, and MLC positions, VMAT significantly reduces treatment time while maintaining or exceeding the dosimetric quality of fixed-field IMRT (24). Navarria et al. (25) studied 341 patients with newly diagnosed high-grade glioma and demonstrated that VMAT achieved better dosimetric conformity and significantly improved mPFS (1.29 vs. 0.99 years, P = 0.02) and mOS (1.56 vs. 1.21 years, P < 0.01) compared to 3D-conformal radiotherapy (3DCRT).

### 2.3 Proton beam therapy

Proton therapy leverages the Bragg peak phenomenon to deposit most of the radiation dose at a defined depth with minimal exit dose. This feature allows superior sparing of healthy brain tissue, making PBT particularly advantageous in pediatric gliomas or recurrent HGGs located near critical structures (26). Several dosimetric and prospective trials suggest reduced neurocognitive decline and lower integral doses with proton therapy, although access remains limited due to high costs and restricted facility availability (27–29).

While clinical evidence for PBT in gliomas is currently limited, its potential appears promising (30). In GBM, due to its aggressive nature and rapid progression, the potential long-term neuroprotective advantages of PBT may be diminished. Future studies should focus on identifying subgroups of patients most likely to benefit from PBT. Younger, functionally independent individuals with high- or low-grade gliomas (HGG or LGG) and favorable molecular profiles may derive more benefit from the reduced normal tissue toxicity associated with PBT. Notably, preliminary small-scale studies in LGG have shown milder acute toxicities with PBT (31).

### 2.4 Carbon-ion radiotherapy

Carbon-ion therapy exhibits a higher relative biological effectiveness (RBE) than photon and proton therapy. RBE, which quantifies biological damage relative to 250 keV X-rays, ranges from 1.1 to 3.74 for carbon ions *in vitro*, depending on cell type (32, 33). Carbon ions induce more complex and lethal DNA damage than photons, with decreased repair efficiency in tumor cells (33–36). One study observed that carbon ion exposure caused pronounced G2/M cell cycle arrest in approximately 79.9% of cells, persisting for at least 48 hours (37, 38). In contrast to photon radiotherapy, where cytotoxicity is dose-dependent, carbon ions appear to exert lethal effects independent of dose duration (39).

In a phase I trial, Qiu et al. (40) enrolled 18 HGG patients to assess the feasibility and safety of carbon ion radiotherapy before proton therapy. Results showed that a pre-proton carbon ion dose of 15 Gy in 3 fractions was well tolerated and potentially beneficial, with a median OS of 17.9 months. No grade ≥3 acute or late toxicities were reported.

### 2.5 FLASH Radiotherapy

FLASH radiotherapy is an emerging technique delivering ultra-high dose rates (>40 Gy/s) and has shown preclinical evidence of reduced normal tissue toxicity while maintaining tumoricidal effects (41). Unlike conventional radiotherapy, which delivers radiation at lower doses over several minutes, FLASH radiotherapy irradiates the tumor in milliseconds. Preclinical studies have shown that FLASH radiotherapy can significantly reduce damage to normal tissues while preserving its tumoricidal effects, making it a promising technique for improving the therapeutic ratio (42). This has potential clinical implications for glioma treatment, as it could reduce neurocognitive side effects typically associated with radiation, while maintaining or improving treatment efficacy. Its application in gliomas remains experimental, but initial murine models demonstrate preserved neurocognitive function compared to conventional dose-rate irradiation (43). Iturri et al. (44) compared proton FLASH radiotherapy (257 ± 2 Gy/s) with conventional-dose-rate proton therapy (4 ± 0.02 Gy/s) in glioma-bearing rats using a single 25 Gy dose. FLASH notably preserved cognitive function and triggered a robust lymphoid immune response in tumors.

### 2.6 Intraoperative radiotherapy

The peritumoral area is a high-risk zone for glioma recurrence. Studies indicate that residual tumor volume significantly increases within the first two weeks post-surgery (45). IORT delivers a single high dose of radiation directly to the tumor bed during surgical resection. This approach minimizes treatment delays and reduces the risk of tumor repopulation between surgery and postoperative radiotherapy (46). Although still investigational in gliomas, early results suggest potential for reducing local recurrence when combined with external-beam RT (47, 48).

## 3 Artificial intelligence in glioma radiotherapy: a transformative role across the treatment spectrum

AI is revolutionizing glioma radiotherapy by enhancing precision, efficiency, and personalization throughout the treatment continuum. From target delineation and treatment planning to imaging biomarker development and prognostic modeling, AI-driven tools are increasingly integrated into clinical workflows (49). These technologies reduce inter-observer variability, automate complex tasks, and offer data-driven insights for individualized treatment strategies. The adoption of radiomics, machine learning, and explainable AI frameworks bridges the gap between imaging data and actionable clinical decisions, ultimately aiming to improve patient outcomes and standardize care.

### 3.1 AI-assisted target delineation

Manual delineation of gross tumor volume (GTV) and clinical target volume (CTV) is time-consuming and prone to inter-observer variability, especially in gliomas with diffuse or infiltrative borders. AI algorithms trained on large imaging datasets have shown high accuracy in auto-segmentation of tumor regions on MRI, reducing variability and standardizing contouring practices. Pehrson et al. (50) reviewed 48 studies and found that AI algorithms showed good concordance with clinicians in GTV delineation across various tumors, with Dice similarity coefficients ranging from 0.62 to 0.92, particularly in encoder–decoder architecture models.

### 3.2 AI in treatment planning

AI is increasingly employed to optimize radiotherapy planning, generating high-quality plans while reducing clinician workload. Knowledge-based planning (KBP) systems use historical treatment data to predict optimal dose distributions, while reinforcement learning models iteratively improve plan quality. These tools enhance conformity indices and spare organs at risk, particularly valuable for gliomas near critical structures such as the optic pathway and brainstem (51).

### 3.3 Radiomics and imaging-based biomarkers

Radiomics transforms standard imaging into high-dimensional, mineable data, enabling extraction of quantitative features that may reflect tumor heterogeneity, infiltration, and treatment response (52). In glioma, radiomics signatures correlate with IDH mutation status, MGMT promoter methylation, and progression patterns. Integrating radiomics with clinical and molecular data enhances risk stratification and supports personalized radiotherapy planning.

### 3.4 Prognostic modeling with machine learning

Machine learning models, including random forests (RF), support vector machines (SVM), XGBoost, and neural networks, have been utilized to predict survival, recurrence, and treatment response in glioma patients (53). Compared to traditional models, these machine learning approaches offer enhanced performance and adaptability in complex clinical scenarios.

#### 3.4.1 RF

Robust and Reliable: RF is an ensemble learning method that combines multiple decision trees to produce highly reliable predictions, reducing the risk of overfitting.

Feature Importance Ranking: RF can identify the most relevant features, providing valuable insights for clinicians to understand the factors influencing prognosis.

Handles High-Dimensional Data: It works effectively with complex datasets like radiomics and genomics, handling large numbers of variables without requiring extensive data preprocessing.

Resistant to Missing Data: RF can handle missing data well, making it robust for clinical datasets with incomplete records (54).

#### 3.4.2 SVM

Effective in High-Dimensional Spaces: SVM excels at handling high-dimensional data, especially when the number of features exceeds the number of samples, making it ideal for radiomics and genomic data.

Strong Generalization: SVM is less prone to overfitting, especially when the data is complex or noisy, and is effective in binary classification tasks.

Clear Margin of Separation: SVM works well when there is a clear boundary between different classes, such as distinguishing between high-grade and low-grade gliomas (55).

#### 3.4.3 XGBoost

High Accuracy: XGBoost is known for its excellent performance, often providing state-of-the-art results in classification and regression tasks.

Handles Missing Data Efficiently: XGBoost can automatically manage missing values, making it ideal for clinical data where missing records are common.

Feature Importance: Like RF, XGBoost provides insights into the importance of different features, which is useful for understanding the key drivers behind predictions.

Scalable and Fast: XGBoost is highly efficient, handling large datasets and providing quick training times, making it scalable for big data applications (56).

#### 3.4.4 Neural networks

Powerful for Complex Data: Neural networks, especially deep learning models, excel at extracting complex patterns from large, high-dimensional datasets like images and multi-omics data.

Adaptable to Various Data Types: They can process a wide range of data types, from structured clinical data to unstructured imaging data, making them versatile in clinical applications.

High Accuracy: When trained on large datasets, neural networks can achieve exceptional accuracy, often outperforming traditional machine learning models (57).

#### 3.4.5 Machine learning applications

Samara et al. (58) developed a classification model using integrated feature selection (Boruta, LASSO, SHAP), identifying 13 key predictors such as IDH1, TP53, and ATRX. XGBoost achieved the highest AUC (0.93), while logistic regression showed the highest testing accuracy (88.09%), with strong model calibration and clinical utility.

Li et al. (59) created an MRI-based radiomics model for high-grade glioma classification using various machine learning algorithms; the Stacking fusion model showed the best performance (AUC = 0.95, sensitivity = 0.84, accuracy = 0.85, F1 score = 0.85). By incorporating imaging, genomic, and clinical data, these models surpass traditional prognostic methods and help identify patients who may benefit from intensified or alternative treatments. Additionally, explainable AI (XAI) frameworks are being developed to enhance transparency and clinical trust in model predictions (60, 61).

## 4 Model interpretability and explainable ai in glioma radiotherapy

One of the major challenges limiting the clinical adoption of AI models in glioma radiotherapy is the lack of interpretability. While AI models, particularly deep learning models, have demonstrated remarkable performance, their black-box nature makes it difficult for clinicians to understand how these models arrive at their predictions. This lack of transparency poses a significant barrier to their acceptance in clinical decision-makingl.

### 4.1 SHAP

SHAP values offer a unified measure of feature importance by quantifying the contribution of each feature to a model’s prediction. In glioma radiotherapy, SHAP can be used to explain which clinical, radiomic, or genomic features have the most significant impact on predicted outcomes, such as patient survival or recurrence. This provides clinicians with valuable insights into the factors driving the model’s predictions (62).

### 4.2 LIME

LIME is another powerful technique that generates interpretable explanations for individual predictions by approximating the AI model with a simpler, interpretable model in the local region around the prediction. This method is particularly useful in radiotherapy when explaining individual patient outcomes, such as why a certain treatment plan is recommended over others (63).

In summary, artificial intelligence holds transformative potential in glioma radiotherapy, providing tools to enhance workflow efficiency and clinical precision. Future directions include multi-omics integration, real-time adaptive planning, and prospective validation of AI models in large-scale clinical trials.
